# Macrophage Checkpoint Nanoimmunotherapy Has the Potential to Reduce Malignant Progression in Bioengineered *In Vitro* Models of Ovarian Cancer

**DOI:** 10.1021/acsabm.4c00076

**Published:** 2024-04-01

**Authors:** Sabrina N. VandenHeuvel, Eric Chau, Arpita Mohapatra, Sameera Dabbiru, Sanjana Roy, Cailin O’Connell, Aparna Kamat, Biana Godin, Shreya A. Raghavan

**Affiliations:** † Department of Biomedical Engineering, Texas A&M University, 3120 TAMU, College Station, Texas 77843, United States; ‡ Department of Nanomedicine, Houston Methodist Research Institute, 6670 Bertner Avenue, Houston, Texas 77030, United States; § School of Engineering Medicine, Texas A&M University, 1020 Holcombe Boulevard, Houston, Texas 77030, United States; △ Division of Gynecologic Oncology, Houston Methodist Hospital, 6550 Fannin Street, Houston, Texas 77030, United States; ⊥ Department of Obstetrics and Gynecology, Houston Methodist Hospital, 6550 Fannin Street, Houston, Texas 77030, United States; ● Houston Methodist Neal Cancer Center, 6445 Main Street, Houston, Texas 77030, United States

**Keywords:** ovarian cancer, metastasis, macrophage, CD47, SIRPα, nanotherapy, immunotherapy

## Abstract

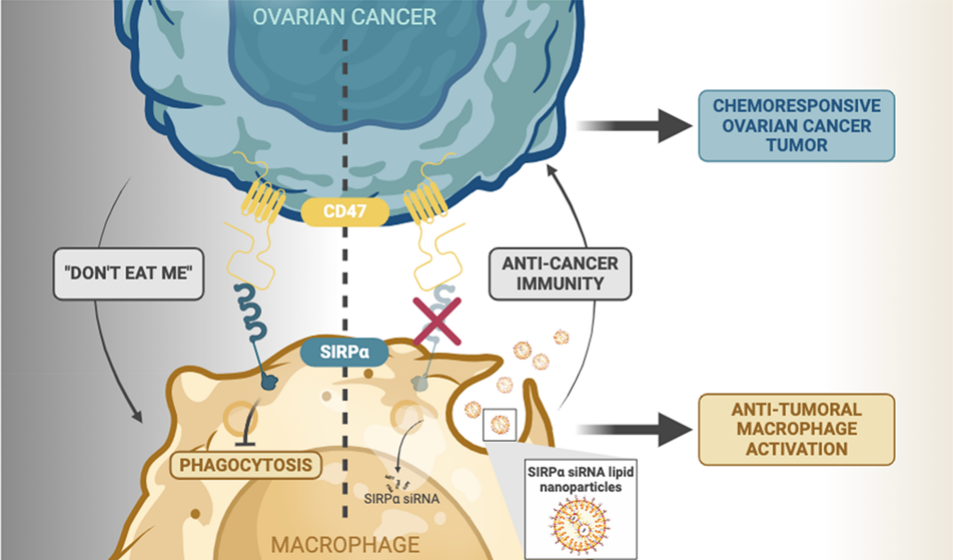

Most ovarian carcinoma (OvCa) patients present with advanced disease at the time of diagnosis. Malignant, metastatic OvCa is invasive and has poor prognosis, exposing the need for improved therapeutic targeting. High CD47 (OvCa) and SIRPα (macrophage) expression has been linked to decreased survival, making this interaction a significant target for therapeutic discovery. Even so, previous attempts have fallen short, limited by CD47 antibody specificity and efficacy. Macrophages are an important component of the OvCa tumor microenvironment and are manipulated to aid in cancer progression via CD47-SIRPα signaling. Thus, we have leveraged lipid-based nanoparticles (LNPs) to design a therapy uniquely situated to home to phagocytic macrophages expressing the SIRPα protein in metastatic OvCa. CD47-SIRPα presence was evaluated in patient histological sections using immunohistochemistry. 3D tumor spheroids generated on a hanging drop array with OVCAR3 high-grade serous OvCa and THP-1-derived macrophages created a representative model of cellular interactions involved in metastatic OvCa. Microfluidic techniques were employed to generate LNPs encapsulating SIRPα siRNA (siSIRPα) to affect the CD47-SIRPα signaling between the OvCa and macrophages. siSIRPα LNPs were characterized for optimal size, charge, and encapsulation efficiency. Uptake of the siSIRPα LNPs by macrophages was assessed by Incucyte. Following 48 h of 25 nM siSIRPα treatment, OvCa/macrophage heterospheroids were evaluated for SIRPα knockdown, platinum chemoresistance, and invasiveness. OvCa patient tumors and *in vitro* heterospheroids expressed CD47 and SIRPα. Macrophages in OvCa spheroids increased carboplatin resistance and invasion, indicating a more malignant phenotype. We observed successful LNP uptake by macrophages causing significant reduction in *SIRPα* gene and protein expressions and subsequent reversal of pro-tumoral alternative activation. Disrupting CD47-SIRPα interactions resulted in sensitizing OvCa/macrophage heterospheroids to platinum chemotherapy and reversal of cellular invasion outside of heterospheroids. Ultimately, our results strongly indicate the potential of using LNP-based nanoimmunotherapy to reduce malignant progression of ovarian cancer.

## 1 Introduction

High-grade serous ovarian carcinoma (OvCa) is a leading cause of cancer-related death in women.^1,2^ The presence of advanced metastatic disease is common at the time of OvCa diagnosis and treatment.^1,3,4^ Despite surgical resection and chemotherapy efforts, patients bearing metastatic lesions experience recurrence rates higher than 80%.^5−8^ The rate of survival for metastatic OvCa has remained effectively stagnant for 40 years,^9^ urging the development of new therapeutic strategies informed by a greater understanding of the metastatic OvCa tumor microenvironment.

Metastatic OvCa cells aggregate within the ascites fluid and form spheroids before seeding secondary organs throughout the peritoneal cavity.^10,11^ In previous work, we established that the hanging drop array methodology closely mimics the presence of OvCa as nonadherent clusters within the ascites.^12−14^ Within the peritoneal ascites fluid, OvCa cells interact with immune and stromal cells (including macrophages among several other cell types) that significantly impact malignant disease progression.^15,16^ Macrophages are the most abundant innate immune cell population in advanced OvCa.^17−19^ Therefore, we refined the use of the hanging drop array to support the growth and interactions of both OvCa cells and macrophages.^20^ Our group and others have shown that macrophages drive OvCa invasion and chemoresistance via a variety of reciprocal signaling mechanisms like Wnt,^20,21^ growth factor^22^ and chemokine^19,23^ secretions, and metabolic crosstalk.^24^

Evaluation of clinical biospecimens links the involvement of macrophage immune checkpoint signaling in OvCa metastatic progression with poor patient prognosis.^21,25,26^ OvCa cells hijack macrophage immune checkpoint signaling by overexpressing self-protein CD47. CD47 binds the macrophage surface ligand SIRPα (signal regulatory protein-α) to trigger a “Don’t Eat Me” signal inhibiting macrophage phagocytosis and permitting OvCa survival. CD47 overexpression is associated with increased migration and invasion at the cancer cellular level and worse prognosis in the clinic. This indicates that the CD47-SIRPα signaling axis is a relevant therapeutic target.^27,28^

CD47-SIRPα signaling has been targeted mainly by anti-CD47 antibodies used in combination with other checkpoint blockades to enhance anticancer function.^29−31^ Incidentally, many cell types in the body aside from just OvCa cells express the self-promoting CD47 protein. Therefore, targeting CD47 as a form of antibody-based immunotherapy has faced the obstacles of both imprecise delivery as well as a high risk of autoimmunity.^32−36^ To address these limitations, we have developed a CD47-SIRPα therapy, which instead aims to reduce SIRPα expression in macrophages and can be delivered more specifically by lipid nanoparticles (LNPs). LNPs have shown tremendous potential and clinical success due to their biocompatibility and biodegradability and have even been previously applied to aid in CD47-SIRPα blockade.^31,35^ LNPs not only prevent cargo degradation in transport but can also be preferentially taken up by phagocytic cells like macrophages, thus specifically homing to these cells.^37−40^ In previous work, we demonstrated that nanotherapy can improve the transport and specificity of therapeutics, specifically those directed toward macrophages in liver metastasis.^41−43^ Thus, our strategy relies on the natural phagocytic ability of macrophages to facilitate LNP uptake and consequently deliver short interfering RNA (siRNA) to knock down SIRPα expression.

In our current work, we test the hypothesis that an LNP system could be used to disrupt CD47-SIRPα immune checkpoint signaling by delivering siRNA to macrophages for metastatic OvCa therapy. Instead of targeting CD47 expression on cancer cells, we exploited the phagocytic ability of macrophages, resulting in a prominent uptake and silencing of SIRPα. This study further demonstrates that siSIRPα LNPs have the ability to overcome macrophage-mediated chemoresistance and reverse malignant OvCa phenotypes in engineered heterospheroids containing OvCa cells and macrophages. The data indicate the potential of the proposed system to limit metastatic OvCa progression and improve patient survival.

## 2 Materials and Methods

### 2.1 Materials

Cell culture reagents and all materials used for Western blotting were purchased from ThermoFisher Scientific (Waltham, MA) unless otherwise specified. Roswell Park Memorial Institute (RPMI) 1640 Medium (Gibco, Grand Island, NY) supplemented with 10% heat-inactivated fetal bovine serum (FBS; Peak Serum, Inc., Wellington CO) and 1X Antibiotic-Antimycotic solution was used as growth medium. Lentivirus encoding the green fluorescent protein (GFP) was obtained from the University of Michigan Vector Core to transduce the OvCa cells.^20^ 1,2-Distearoyl-*sn*-glycero-3-phosphocholine (DPSC) and 1,2-distearoyl-*sn*-glycero-3-phosphoethanolamine-N-[methoxy (polyethylene glycol)-2000] (ammonium salt) (DSPE-PEG(2000)) were purchased from Avanti Polar Lipids (Alabaster, AL); cholesterol from Sigma-Aldrich (St. Louis, MO); 4-(dimethylamino)-butanoic acid, (10Z,13Z)-1-(9Z,12Z)-9,12-octadecadien-1-yl-10,13-nonadecadien-1-yl ester (MC3) from MedChemExpress (Monmouth Junction, NJ); and citrate buffer from ThermoFisher Scientific.

### 2.2 SIPRα-Dependent Patient Survival

Survival analysis was conducted using data publicly available on the University of California—Santa Cruz (UCSC) Xena Browser.^44^ The Cancer Genome Atlas Ovarian Cancer data set (TCGA OV) was analyzed to categorize patients into high and low *SIPRα* expression, where expression levels in the top 10% were considered high (9 of 278 patients). Categorized disease-specific survival data was exported and plotted as a Kaplan–Meier survival curve in GraphPad Prism.

### 2.3 Histological Staining of Patient Tissues for SIPRα and CD47 Expression

Primary patient tumor tissues were collected from consenting patients (IRB #PRO00029517, MOD00005725, Houston Methodist Hospital). Pathology evaluation demonstrated histologic findings consistent with high-grade serous OvCa. Tissue processing, sectioning, and staining were performed at the Houston Methodist Pathology Core. Paraffin-embedded tumor samples were sectioned and deparaffinized using xylene and ethanol washes. Heat-mediated antigen retrieval was completed with Tris-EDTA (pH 9.0). Slides were incubated with hydrogen peroxide to eliminate endogenous peroxidase activity and horse serum blocking buffer to reduce nonspecific binding of antibodies. Primary monoclonal antibodies for CD47 (1:100) or SIRPα (1:250; Novus Biologicals, Littleton, CO) were incubated with samples overnight at 4 °C followed by a 30 min incubation with secondary antibodies at room temperature. DAB Substrate-Chromogen was allowed to incubate until the appropriate color change was visualized compared to positive control samples. Slides were counterstained with Hematoxylin. Additionally, the slides were subjected to standard hematoxylin and eosin processing. A BZ8000 microscope (Keyence, Osaka, Japan) was used to acquire bright-field images of samples.

### 2.4 Cell Culture

The high-grade serous OvCa cell line, OVCAR3, and THP-1 monocytes were purchased from the American Type Culture Collection (ATCC; Manassas, VA). They were maintained in complete RPMI culture medium at 37 °C with 5% CO_2_. OvCa cells were transduced with lentiviral GFP following previously established protocols.^20^ Cells were grown in traditional 2D culture until seeding into 3D spheroids (see Spheroid Formation on 384-Well Hanging Drop Arrays). Monocytes were differentiated into M0 macrophages using 1 ng/mL phorbol-12-myristate-13-acetate (PMA; Sigma-Aldrich) for 72 h on hanging drop spheroid arrays.^20^

### 2.5 Spheroid Formation on 384-Well Hanging Drop Arrays

OvCa monospheroids or OvCa/macrophage heterospheroids were formed on a 384-well hanging drop array following well-established protocols.^12−14,20,45,46^ OvCa monospheroids were initiated with 100 cells in a 20 μL drop of growth medium. To create OvCa/macrophage heterospheroids, the hanging drop platform was first leveraged to differentiate THP-1 monocytes to M0 macrophages by phorbol ester exposure.^20^ Following a 3 day differentiation, macrophages were harvested and mixed with OvCa cells (50:50) to create heterospheroids. Monospheroid or heterospheroid formation was visually confirmed by using phase contrast microscopy at day 4.

### 2.6 Flow Cytometry

OvCa/macrophage heterospheroids were harvested for flow cytometry on day 4. Spheroids were mechanically dissociated into single-cell suspensions in phosphate buffered saline (PBS) supplemented with 2% FBS. Cells were incubated with CD47 or SIRPα antibodies tagged with AlexaFluor 647 (BD Biosciences, Haryana, India) for 30 min at 37 °C. Isotype controls were used to establish background staining for AlexaFluor 647. After washing away unbound antibody and resuspending in fresh buffer, flow cytometry analysis was performed using the Attune NxT (ThermoFisher Scientific). Unstained and isotype controls were employed to establish a gating strategy, cutting off a background gate at 0.5%. The percentages of cells expressing CD47 or SIRPα were determined in the OvCa/macrophage heterospheroids.

### 2.7 Immunofluorescence Staining

Cells were fixed for imaging with a 4% paraformaldehyde (PFA) solution. Fixed cells were permeabilized with 1% Triton X-100 (Sigma-Aldrich) and blocked with 10% goat serum. Fluorophore-conjugated primary antibodies (SIRPα, AlexaFluor 647) were added to cells at a 1:100 dilution for an overnight incubation after which unbound antibodies were rinsed off with PBS. Nuclei were counterstained with DAPI, and fluorescence was visualized on an SP8 confocal microscope (Leica Microsystems, Wetzlar, Germany).

### 2.8 Western Blot SIRPα Protein Identification

Protein was extracted from heterospheroids with RIPA buffer and quantified using the Pierce BCA Protein Assay Kit. 20 μg of protein was loaded into each lane of a polyacrylamide gel and transferred to a low-fluorescence PVDF transfer membrane along with the PageRuler Prestained Protein Ladder (10–180 kDa). The membrane was blotted with a SIRPα polyclonal antibody as well as β-Actin for the loading control. A horseradish peroxidase (HRP)-conjugated secondary antibody (rabbit antihuman IgG; Invitrogen) was used to visualize bands on the LI-COR C-Digit 3600 Western Blot Scanner (LI-COR Biosciences, Lincoln, NE).

### 2.9 MTS Cell Proliferation Assay

An MTS assay (Abcam, Cambridge, UK) was used to assess cell proliferation over time. Spheroids were evaluated at days 0, 2, 4, and 6 to generate paired samples. At the time point of interest, cells were incubated with the MTS reagent diluted 1:10 in each 20 μL hanging drop of culture medium at 37 °C. After a 2 h incubation, absorbance was measured at 490 nm with the Cytation 7 microplate reader (BioTek, Winooski, VT). Reported values were normalized to a day 0 absorbance measurement for a time-dependent proliferation analysis.

### 2.10 Response to Chemotherapy

Carboplatin (MedChemExpress, Monmouth Junction, NJ) was dissolved by sonication in sterile PBS at a stock concentration of 5 mg/mL. OVCAR3 monospheroids or OVCAR3/macrophage heterospheroids formed over 4 days were treated with 0–500 μM carboplatin and incubated for 2 days/48 h. An MTS assay was then used to determine the percent cell viability in carboplatin-treated spheroids compared with untreated controls. IC_50_ values were calculated with the GraphPad Prism normalized log(inhibitor) variable slope nonlinear fit function.

### 2.11 SIRPα siRNA (siSIRPα) LNP Design

siSIRPα LNPs were formulated using the Nanoassemblr Benchtop (Precision NanoSystems, Vancouver, BC, Canada) by mixing the ethanolic phase containing DPSC:DSPE-PEG2000:cholesterol:MC3 at the molar ratio 8:1.5:38.5:52 with the aqueous phase containing 50 μg/mL siSIRPα in 0.1 M citrate buffer (pH 5). One volume of ethanolic phase was mixed with three volumes of aqueous phase at a combined flow rate of 12 mL/min. Residual ethanol and unencapsulated siRNA were removed from the solution with two dialyses in PBS (>4 h each) using a D-Tube Dialyzer Maxi (12–14 kDa, Sigma-Aldrich). The systems were diluted in PBS to a final 500 nM working concentration.

### 2.12 siSIRPα LNP Characterization

The resulting siSIRPα LNPs were characterized for particle diameter, polydispersity index (PDI), and zeta potential by dynamic light scattering (DLS; Malvern Zetasizer, Malvern Instruments, Malvern, UK) and for RNA content using the RiboGreen RNA Assay (ThermoFisher Scientific) according to the manufacturer’s instructions. Encapsulated and unencapsulated RNA were quantified. LNPs were digested with 1% Triton X-100 to release RNA contents for quantification of total (encapsulated + unencapsulated) RNA. The encapsulation efficiency (EE) was calculated based on the formula
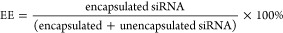


For reproducibility, 3 different batches were prepared and tested. LNPs were stored at 4 °C for up to 10 days after preparation at the final working concentration of 500 nM. Stability (size, PDI, and zeta potential) was assessed daily over those 10 days. LNP Tracking Analysis (NTA) was utilized to quantify particle concentration at days 1 and 10 using a NanoSight NS300 instrument (Malvern Panalytical, Malvern, UK). Particle samples were diluted (1:10,000) in nuclease-free water and measured for 30 s with manual shutter and gain adjustments on the 532 nm (green) laser. Three measurements were taken for each of the 3 samples and analyzed with NTA 3.4 Build 3.4.4 software.

To confirm the LNP ultrastructure, transmission electron microscopy (TEM) was performed at the Texas Heart Institute CV Pathology Core Electron Microscopy Laboratory. siRNA LNPs (8–10 μL) were applied on a Formvar/carbon-film coated mesh grid. Excess solution was blotted with filter paper, and the samples were allowed to air-dry for 10 min at room temperature. Uranyl Acetate solution (0.2%; Sigma-Aldrich) was used as a negative stain. The samples were imaged under a JEOL 1230 transmission electron microscope (JEOL, Ltd., Tokyo, Japan) at 2–5 × 10^4^ magnification.

### 2.13 LNP Uptake by Macrophages

LNP uptake was assessed in naïve M0 macrophages. THP-1 monocytes were seeded at 4,000 cells/well in 96-well culture plates with 1 ng/mL PMA for 3 days. To monitor the uptake capabilities of macrophages, siSIRPα LNPs were fluorescently tagged with 1,1′-dioctadecyl-3,3,3′,3′-tetramethylindocarbocyanine perchlorate (DiL; ThermoFisher, Scientific). 25 nM siSIRPα LNPs were added, and cells were maintained for 48 h with hourly imaging on the Incucyte Live Cell Analysis system. Red fluorescence and phase images (10×, 5 images/well) were taken at each time point and analyzed using the Incucyte Live Cell Image Analysis software for average relative fluorescence units, per well, over time.

### 2.14 RNA Extraction and qPCR

RNA was extracted from adherent macrophages or harvested heterospheroids with the RNeasy Mini Kit (Qiagen, Hilden, Germany). For gene expression analysis of invasiveness, a fluorescence activated cell sorting step was added to separate OVCAR3 cells cultured in heterospheroids with macrophages. The GFP tag on the OVCAR3 cells was used to separate the OvCa cells from macrophages on either a Beckman Coulter Moflo Astrios (Beckman Coulter, Brea, CA) or BDFACS Aria II (BD Biosciences) Cell Sorter at the Veterinary Pathobiology Flow Cytometry Facility and School of Medicine Analytical Cytometry Core, respectively, at Texas A&M University.

RNA concentration and purity were measured with a NanoDrop OneC (ThermoFisher Scientific). RNA was converted to cDNA with an Applied Biosystems High Capacity cDNA Reverse Transcription Kit. qPCR was then performed using an SYBR Green PCR MasterMix (ThermoFisher Scientific) on a QuantStudio 3 Real-Time PCR System (Applied Biosystems). Gene expression changes were calculated using the 2ΔΔC_t_ method with *GAPDH* as a housekeeping gene. Gene primer sequences are listed in Table 1.

**Table 1:** Primer Sequences for Genes Analyzed with qPCR

Gene	Forward primer sequence	Reverse primer sequence
*GAPDH*	CTGGGCTACACTGAGCACC	AAGTGGTCGTTGAGGGCAATG
*SIRPα*	GGCCTCAACCGTTACAGAGAA	GTTCCGTTCATTAGATCCAGTGT
MMP-9	TGTACCGCTATGGTTACACTCG	GGCAGGGACAGTTGCTTCT
*SNAI1*	TCGGAAGCCTAACTACAGCGA	AGATGAGCATTGGCAGCGAG
*ZEB1*	GATGATGAATGCGAGTCAGATGC	ACAGCAGTGTCTTGTTGTTGT
*NOS2*	TTCAGTATCACAACCTCAGCAAG	TGGACCTGCAAGTTAAAATCCC
*IL-12*	CCTTGCACTTCTGAAGAGATTGA	ACAGGGCCATCATAAAAGAGGT
*IL-1β*	ATGATGGCTTATTACAGTGGCAA	GTCGGAGATTCGTAGCTGGA
*IL-10*	GACTTTAAGGGTTACCTGGGTTG	TCACATGCGCCTTGATGTCTG
*CD206*	GGGTTGCTATCACTCTCTATG	TTTCTTGTCTGTTGCCGTAGTT

### 2.15 siSIRPα LNP Immune Checkpoint Treatment

Monospheroids or heterospheroids were treated with 25 nM siSIRPα LNPs on day 2 of hanging drop culture. The LNPs’ ability to reduce both macrophage *SIRPα* expression and OvCa metastatic behavior was evaluated over 4 more days.

### 2.16 Macrophage-Mediated OvCa Invasive Potential

Monospheroids or heterospheroids treated with LNPs or maintained as controls were seeded into 96-well culture plates following 4 days in hanging drop culture. Stacked phase contrast micrographs were taken of each spheroid over 5 days to monitor cell migration as a measure of invasiveness. The StackFocuser plugin on the NIH ImageJ software was used to compress each image stack into a single focused 2D plane. Spheroid perimeters were drawn by hand in ImageJ to measure spheroid area across various time points. Fold change in area was calculated based on day 0 images of spheroids immediately after transfer from hanging drop culture. Increased spheroid area was used to track cell migration over time, indicating OvCa invasive potential.

### 2.17 Statistical Analysis

GraphPad Prism 10 software was used to perform statistical analysis and hypothesis testing. All resulting values are reported as the mean and standard error of the mean. Spheroid-based experiments were conducted with 3–6 biological replicates containing 10–25 technical replicates (spheroids) each. Drug response IC_50_ values were generated by normalizing MTS-mediated absorbance values to untreated controls. Similarly, fold changes in spheroid proliferation and migration were calculated by normalizing absorbance values to a day 0 control for each condition. All qPCR data was compared to control conditions within the experiment and performed in triplicate with 3 biological replicates.

## 3 Results

### 3.1 CD47-SIRPα Axis Is Clinically Significant in Ovarian Cancer

**Figure 1:**
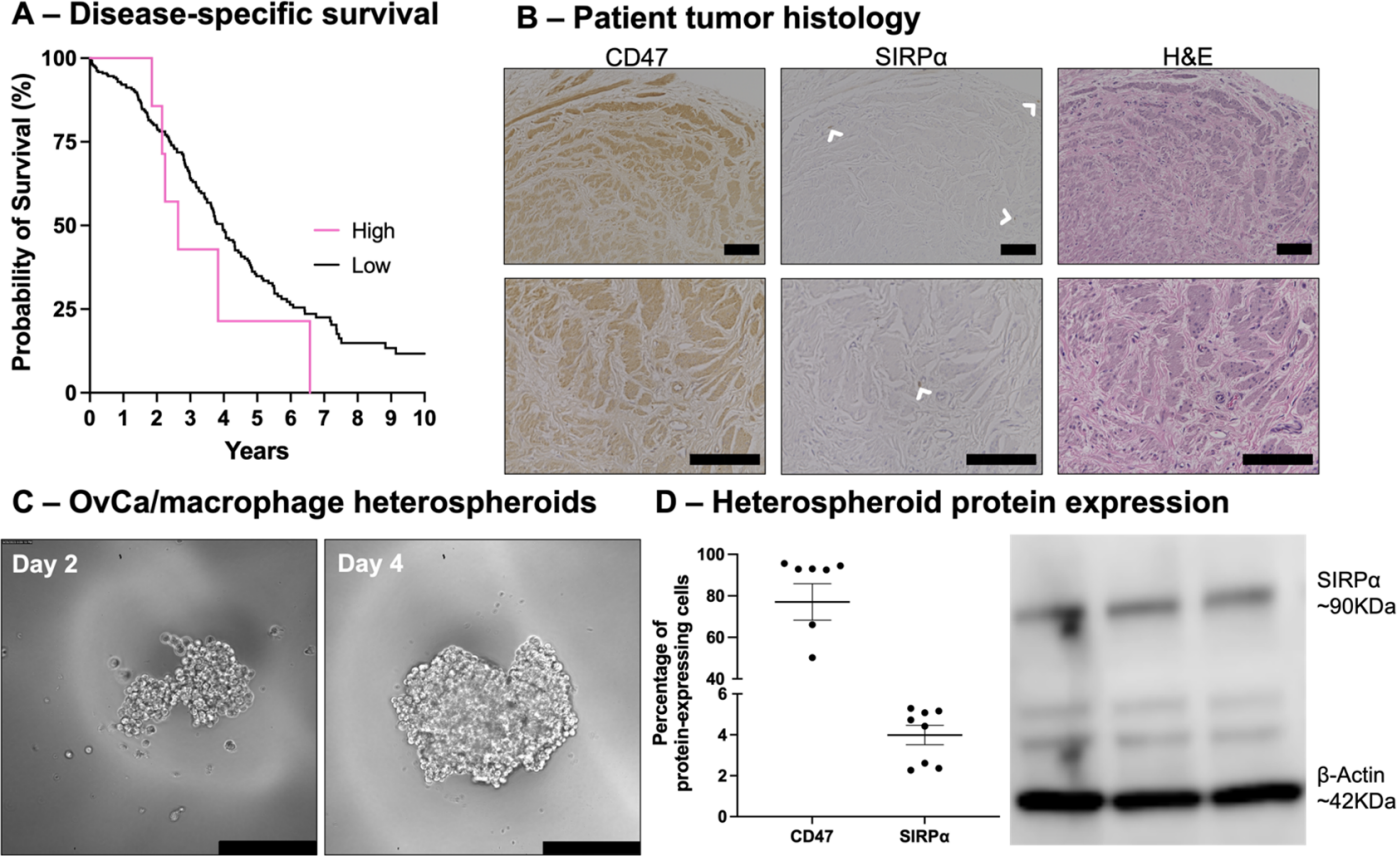
CD47-SIRPα presence in ovarian cancer. (A) Kaplan–Meier survival curve generated from the UCSC Xena Browser. Median survival for patients with SIRPα expression in the top 10% decreases by 1.5-fold (*p* = 0.25, log-rank test). (B) Histological staining of primary patient OvCa tumor samples. Brightfield images (20× and 40×) show brown color change in the slides indicating the presence of CD47 (left) and SIRPα (middle) with white arrows indicating SIRPα^+^ cells. H&E stains (right) illustrate overall tissue structure. Scale bars = 100 μm. (C) Phase contrast micrographs of spheroids generated from 50 OVCAR3 cells and 50 THP-1-derived M0 macrophages. By day 2, cells began to aggregate. Cells proliferated to form a compact spheroid by day 4. Scale bars = 200 μm. (D) Flow cytometry analysis (left) of OvCa/macrophage heterospheroids confirmed 77.05 ± 8.76% of cells within the spheroids express CD47 and 3.99 ± 0.47% express SIRPα. Western blotting (right) further validates the maintenance of a SIRPα^+^ macrophage population within the OvCa/macrophage heterospheroids with the presence of a band at ∼90 kDa.

The CD47 and SIRPα proteins are negatively associated with OvCa progression, making them highly relevant targets for therapeutic discovery. Specifically, median disease-specific survival is reduced by 1.5-fold in cases of high (top 10%) *SIRPα* expression in OvCa patients (*p* = 0.25, log-rank test, Figure 1A). Primary patient tumors from a high-grade serous patient express both CD47 and SIRPα, as evidenced by the presence of the brown immunohistochemistry stain (Figure 1B), further indicating the potential to study and target this macrophage immune checkpoint in the context of OvCa progression.

Considering the correlation between patient outcome and macrophage checkpoint signaling in clinical OvCa, we utilized a heterospheroid model to study these interactions *in vitro*. OVCAR3 cells were seeded on the hanging drop array with THP-1-derived M0 macrophages to create heterospheroids. The aggregation of OVCAR3 and M0 macrophages into a heterospheroid entity is visualized at days 2 and 4 (Figure 1C). Following 4 days of spheroid formation, flow cytometry analysis showed that 77.05 ± 8.76% of cells in heterospheroids expressed CD47 and 3.99 ± 0.47% of cells expressed SIRPα (Figure 1D). Presence of the SIRPα protein within the OvCa/macrophage heterospheroids was also confirmed by Figure 1 immunoblotting (presence of a band at ∼90 KDa; Figure 1D).

### 3.2 Macrophages Impact OvCa Growth and Chemoresistance

**Figure 2:**
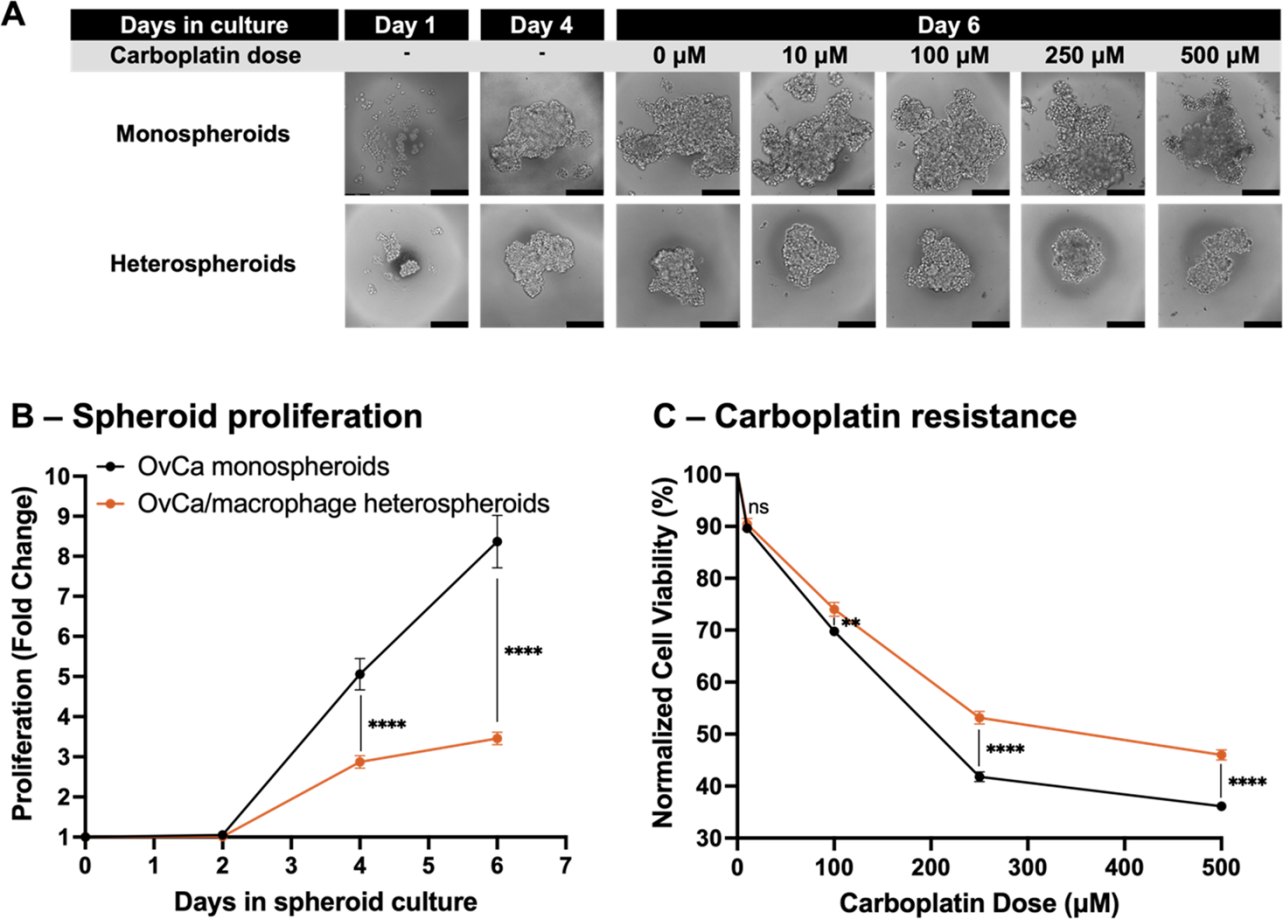
Macrophage-dependent chemoresistance in 3D spheroids. (A) Phase contrast micrographs of monospheroids (OvCa cells alone) and heterospheroids (OvCa/macrophage co-culture). Comparison of day 1 and 4 images shows the cells clustering at the bottom of the hanging drop and forming a single compact spheroid by day 4. Mono- and heterospheroids were seeded with 100 cells/drop (day 0), but monospheroids appear larger by day 4. Compact spheroids were treated with carboplatin chemotherapy (0–500 μM) on day 4 and visualized at day 6. Cell death was evident by increased cellular debris surrounding the spheroid as well as less defined spheroid and cell boundaries. Scale bars = 200 μm. (B) Proliferation measured by MTS viability assay confirmed the increased sizes observed in monospheroids compared to heterospheroids in spheroid images. Monospheroids grew nearly 2.4 times more than heterospheroids by day 6 owing to the terminal differentiation state of macrophages which inhibited their proliferation. (C) Monospheroid and heterospheroid response to carboplatin chemotherapy (0–500 μM) was measured with an MTS viability assay. Heterospheroids were more resistant demonstrated by significantly higher viability at doses of 100–500 μM carboplatin (4.23% increase at 100 μM, **p < 0.01; 11.37% at 250 μM and 9.87% at 500 μM, ****p < 0.0001, two-way ANOVA). Heterospheroid viability data also produced a higher IC_50_ value of 368.2 μM compared to that of monospheroids (218.1 μM, ns, one-way ANOVA).

Spheroids formed from 100 OvCa cells (monospheroids) or 50 OvCa with 50 macrophages (heterospheroids) were cultured for 6 days for analysis of proliferation over time. By day 4, a single compact spheroid could be seen with defined margins (Figure 2A). On days 4 and 6, MTS viability measurements were compared to those taken at the time of spheroid initiation (day 0) to produce a fold change in proliferation over time. Despite seeding 100 cells/drop in both conditions (day 0), monospheroids proliferated faster than heterospheroids, showing 5.06 ± 0.39-fold and 8.37 ± 0.66-fold changes at days 4 and 6 compared to 2.87 ± 0.16-fold and 3.46 ± 0.16-fold in heterospheroids, respectively (****p < 0.0001, two-way ANOVA, Figure 2B). Visual confirmation of cell proliferation can be observed in phase contrast micrographs, where mono- and heterospheroids are similarly compact in structure, but monospheroids appear larger in cell number (Figure 2A).

Compact spheroids were treated on day 4 with the platinum-based chemotherapy drug carboplatin (0–500 μM). Following 48 h of incubation with the drug, viability was determined using the MTS assay, from which IC_50_ values for carboplatin were determined. Carboplatin treatment decreased cell viability in both OvCa monospheroids and OvCa/macrophage heterospheroids. This was visually apparent at day 6 (2 days following carboplatin treatment), where cell death can be observed through less defined spheroid boundaries surrounded by increased amounts of cellular debris (Figure 2A, day 6). OvCa monospheroids appeared to be more susceptible to chemotherapy treatment based on these visual cues than heterospheroids (Figure 2A, day 6). Shifts in viability curves were quantified and are reported in Figure 2C. Heterospheroids were, in fact, more resistant to carboplatin as they maintained 4.23% higher viability than monospheroids after 100 μM (**p < 0.01, two-way ANOVA, Figure 2C) and 11.37% and 9.87% following 250 and 500 μM, respectively (****p < 0.0001, two-way ANOVA, Figure 2C).

### 3.3 Macrophage Checkpoint Nanoimmunotherapy Design and Characterization

**Figure 3:**
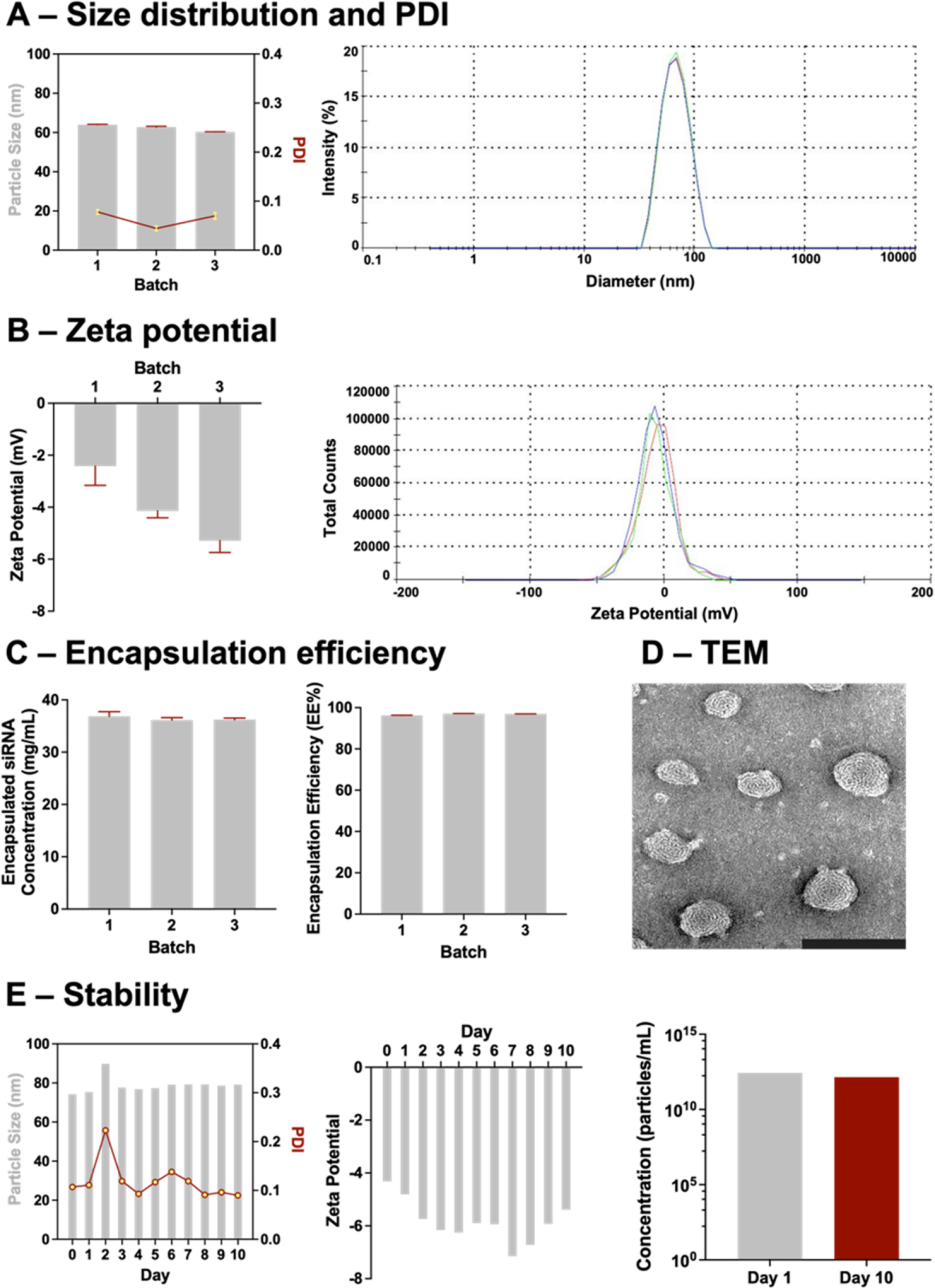
LNP design and characterization. LNPs were fabricated using microfluidic techniques to encapsulate siSIRPα and were characterized for stability and consistency. (A) siSIRPα LNPs from 3 separate prepared batches measured around 60 nm in diameter with low PDI (<0.1) indicating fabrication consistency both within and between batches of particles as measured by DLS (left). Size distribution plots also demonstrate the desired consistency in particle diameter (right). (B) DLS analysis showed siSIRPα LNPs were neutrally charged (zeta potential −4 mV, left) with consistent distribution between batches (right). (C) The amount of siRNA encapsulated within the particles remained highly consistent between batches (left) with better than 95% efficiency in encapsulating siRNA in all batches (right). (D) TEM of siSIRPα LNPs. Particle size distribution can be visualized in the TEM taken at 20,000× magnification. Scale bar = 200 nm. (E) Particle size and PDI (left), zeta potential (middle), and encapsulation (right) remain consistent over 10 days indicating the stability of the siSIRPα LNPs for the duration of experiments.

Major obstacles to RNA therapies include nucleic acid stability and off-target effects or premature drug clearing. To improve transport and specificity of SIRPα siRNA (siSIRPα) as a macrophage-targeted immunotherapy, lipid nanoparticles (LNPs) were designed to encapsulate and deliver the siRNA. siSIRPα LNPs were evaluated for particle size, polydispersity index (PDI), zeta potential, encapsulation efficiency (EE), and RNA concentration. Reproducibility and stability were assessed for 3 separate batches prepared at least 1 week apart and tracked over 10 days. The particles were formulated with an average diameter of 62.32 ± 0.56 nm (Figure 3A). The LNPs exhibited very uniform size distribution and PDI (<0.1, Figure 3A). Zeta potential also remained consistent at −3.95 ± 1.45 mV (Figure 3B). The concentration of encapsulated siRNA was 36.41 ± 0.39 μg/mL, and the EE was greater than 95% (Figure 3C). LNP size, PDI, zeta potential, RNA contents, and encapsulation were very reproducible with less than 10% variation between the batches for all measured criteria. These values also remained constant over 10 days, confirming the stability of the LNPs over the course of our experiments (Figure 3E). TEM of the siSIRPα LNPs confirmed the uniform size distribution as well as the bilayer structure of the delivery system (Figure 3D).

### 3.4 siSIRPα LNP Uptake and Alteration of Macrophage Phenotype

**Figure 4:**
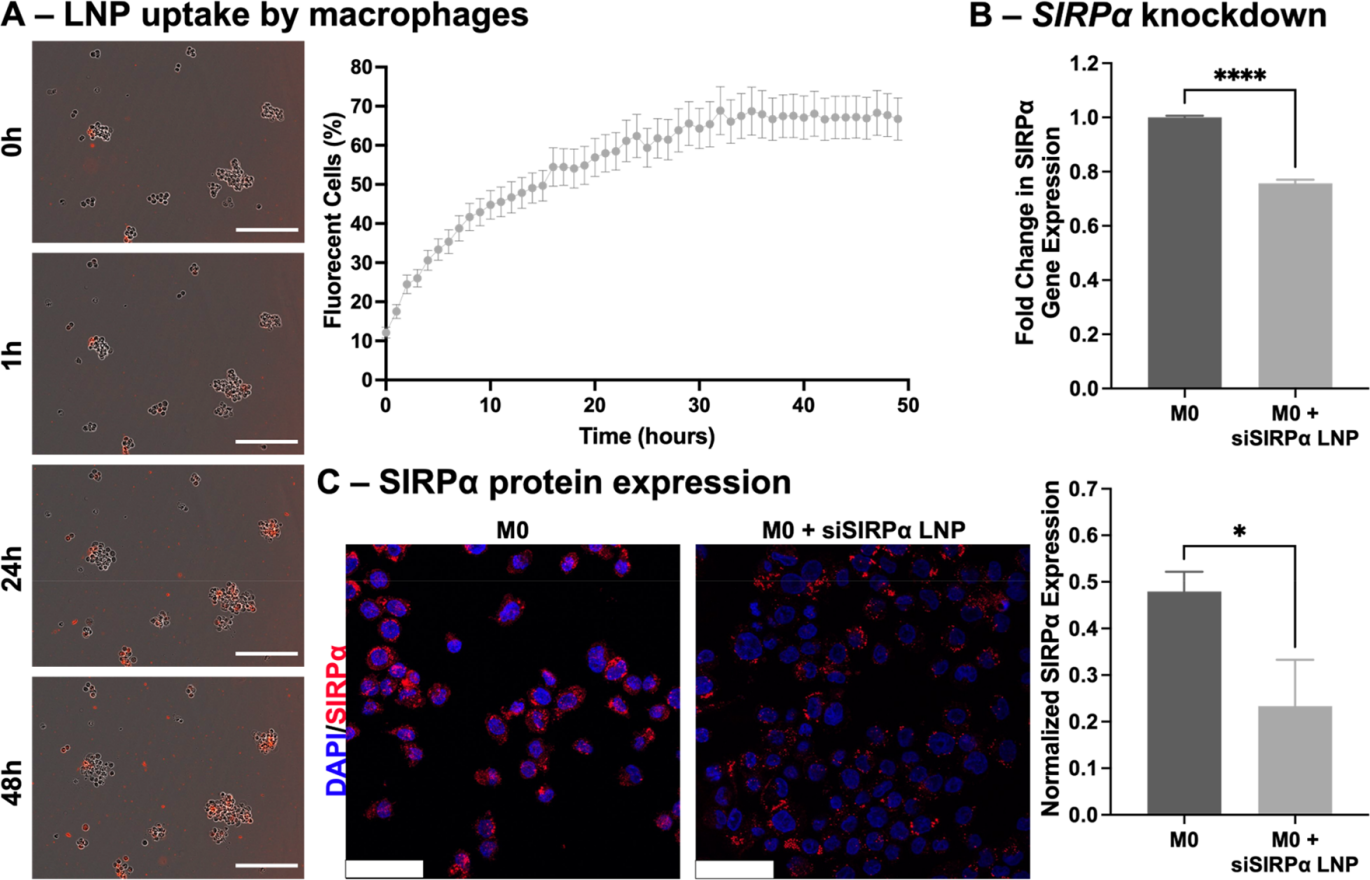
siSIRPα LNP uptake and alteration of macrophage phenotype. (A) Live-cell imaging of M0 macrophages in a 2D culture. Fluorescently labeled LNPs containing SIRPα siRNA were administered to macrophages and monitored over 48 h with hourly imaging. Macrophages can be visualized in the brightfield images overlaid with red fluorescence indicating LNP uptake which visibly increased over time as more macrophages phagocytosed the LNPs (left, scale bars = 200 μm). Analysis of hourly images over 48 h showed more than 50% of cells uptake the LNPs by 30 h (right). (B) Gene expression analysis indicated that macrophages treated with siSIRPα LNPs expressed 24.28 ± 1.34% less SIRPα than untreated controls (****p < 0.0001, unpaired *t* test). (C) Knock down of the *SIRPα* gene translated to 49% less SIRPα protein expression evidenced by representative immunofluorescence images comparing siSIRPα LNP-treated M0 macrophages (normalized fluorescence intensity 0.23 ± 0.10) to untreated controls (normalized fluorescence intensity 0.48 ± 0.04, *p < 0.05, one-way ANOVA). Scale bars = 50 μm.

*In vitro* kinetic studies were conducted to test LNP uptake by macrophages using naïve M0 THP-1 monocyte-derived macrophages. Macrophage uptake of red fluorescent LNPs was evident by the increase in red fluorescence over 48 h (Figure 4A). Naïve M0 macrophages were able to efficiently engulf siSIRPα LNPs as visualized in images of fluorescently labeled particles. As expected, macrophage engulfment of LNPs started immediately upon their addition to macrophage cultures (Figure 4A, 1 h). By 30 h, more than 50% of cells showed red LNP-associated fluorescent signal indicating particle uptake (Figure 4A). Even alternatively activated macrophages (M2-like) had robust uptake of siSIRPα LNPs (Figure S1A,B).

Macrophage uptake of the siSIRPα LNPs reduced the gene expression of *SIRPα*. Following treatment, LNPs caused a 24.28 ± 1.34% knockdown in M0 macrophages differentiated from THP-1 monocytes (****p < 0.0001, unpaired *t* test, Figure 4B). Importantly, knockdown of the *SIRPα* gene translated to a 49% reduction in SIRPα protein expression by immunofluorescence staining (*p < 0.05, one-way ANOVA, Figure 4C) indicating the potential for siSIRPα LNPs to cause functional changes in the relationship between OvCa cells and macrophages.

### 3.5 siSIRPα LNP Efficiency in OvCa/Macrophage Heterospheroids

**Figure 5:**
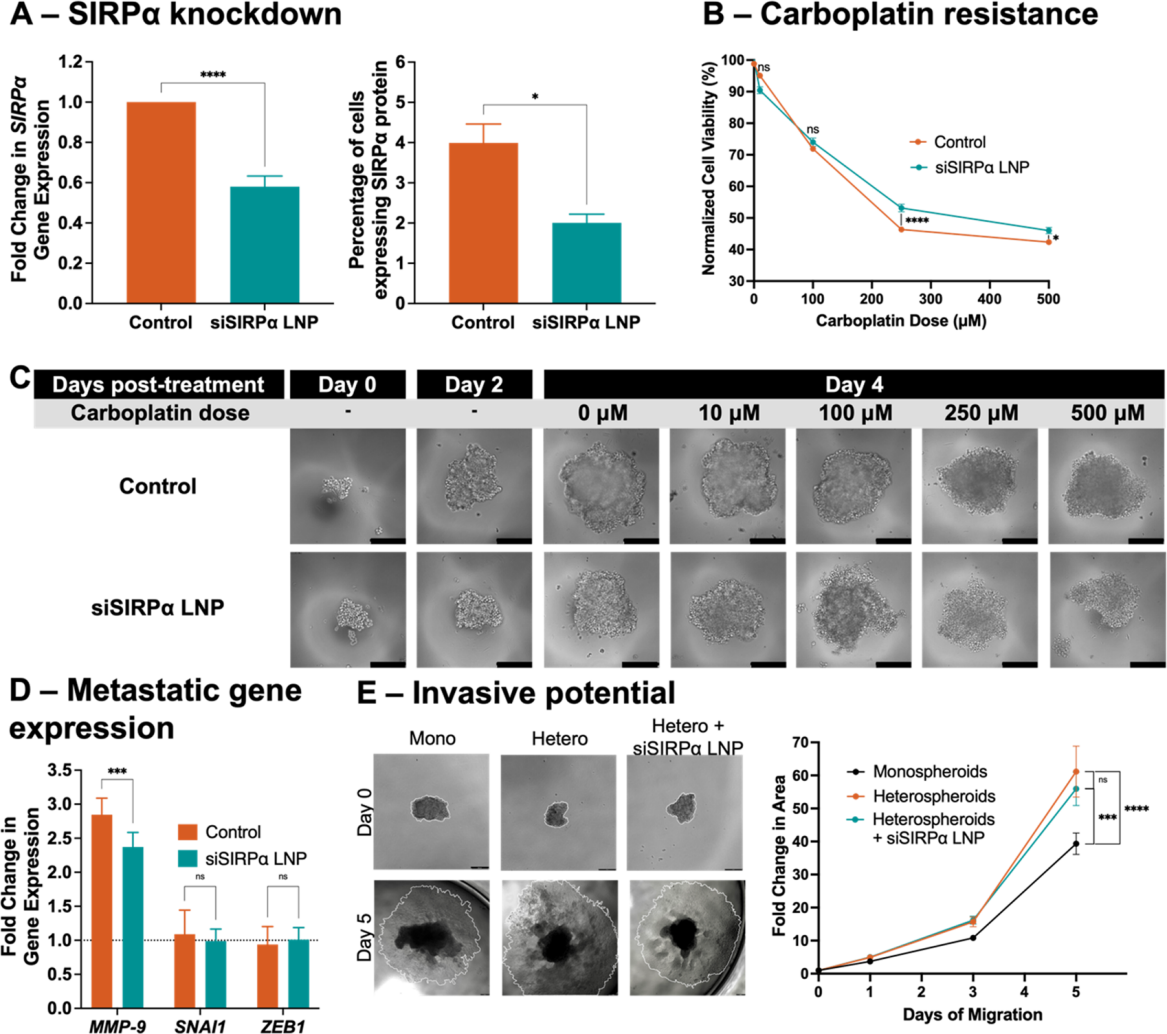
Reducing *SIRPα* reduces metastasis-indicating behavior in OvCa/macrophage heterospheroids. (A) siSIRPα LNP treatment of heterospheroids resulted in a reduction of nearly 42% of *SIRPα* gene expression (****p < 0.0001, unpaired *t* test, left) which translated to a similar 50% reduction in the SIRPα surface protein measured by flow cytometry (*p < 0.02, unpaired *t* test, right). (B) Carboplatin chemotherapy treatment (0–500 μM) revealed that this decrease in *SIRPα* expression was linked to a recovery of the chemotherapy response in resistant heterospheroids. In fact, siSIRPα LNPs induced a 32% recovery from the resistance caused by macrophages as compared to the control IC_50_ of the OvCa monospheroid (ns, one-way ANOVA). At 250 and 500 μM carboplatin doses, LNP-treated spheroids showed 6.79% and 3.64% improvement in chemotherapy response (****p < 0.0001, *p < 0.05, respectively, two-way ANOVA). (C) Phase contrast micrographs of control and LNP-treated heterospheroids. Images showed slowed growth by day 2 following LNP treatment. LNP-treated heterospheroids were more sensitive to carboplatin chemotherapy, showing increased cell death by day 4 across 5 doses used to generate an IC_50_ value. Scale bars: 200 μm. (D) Metastasis-indicating gene analysis of OvCa cells. Cells from heterospheroids were treated with siSIRPα LNPs or maintained as controls and sorted to analyze the OvCa cells alone. Both heterospheroid conditions were compared to macrophage-naïve OVCAR3 cells from monospheroids. Loss of *MMP-9* gene expression was observed after siSIRPα LNPs (compare 2.85-fold increase in MMP-9 in control heterospheroids to 2.37-fold after LNPs, ***p = 0.0005, two-way ANOVA) which indicated OvCa cells becoming less invasive following LNP treatment. Similarly, there is a small decrease in epithelial-to-mesenchymal transition gene *SNAI1* (ns, two-way ANOVA). (E) Spheroid migration model created to measure OvCa invasiveness. Spheroids were maintained in hanging drop culture for 4 days with siSIRPα LNP treatment on day 2. After seeding onto 2D well plates, cellular migration was tracked via changes in area occupied by cells as they traveled away from the spheroid. This area is indicated by the white outlines drawn in ImageJ software (left, scale bars = 200 μm). Macrophage presence induced a significant increase in migratory ability (right, ****p < 0.0001, two-way ANOVA) which was partially reversed in spheroids which were pretreated with LNPs prior to seeding (ns, two-way ANOVA).

Heterospheroids formed from OVCAR3 cells and THP-1-derived macrophages were treated with 25 nM siSIRPα LNPs. After 48 h of incubation with the LNPs, gene and protein expression were quantified to evaluate the nanoimmunotherapy efficiency in reducing SIRPα expression in an OvCa/macrophage heterospheroid setting. LNP treatment produced a 41.97 ± 5.25% knockdown in *SIRPα* gene expression compared to untreated controls (****p < 0.0001, unpaired *t* test, Figure 5A). Further characterization was provided by flow cytometry analysis which showed similar reduction of SIRPα expression in heterospheroids upon LNP treatment (compare 3.99 ± 0.47% of untreated to 2.01 ± 0.22% of LNP-treated cells expressing the SIRPα surface protein, *p < 0.02, unpaired *t* test, Figure 5A).

### 3.6 siSIRPα LNPs Functionally Manipulate OvCa Metastatic Behavior

After confirming the knockdown efficiency of the siSIRPα LNP immunotherapy, we tested functional repercussions of interfering with “Don’t Eat Me” signaling between OvCa cells and macrophages within heterospheroids. We evaluated this by quantifying changes in OvCa response to carboplatin chemotherapy as well as metastasis-indicating gene expression and invasive potential.

siSIRPα LNP treatment increased heterospheroid sensitivity to carboplatin, first visually apparent by the loss of heterospheroid integrity under carboplatin-treated conditions that also received LNP treatment (Figure 5C, day 4). Quantification of cell viability subsequently demonstrated that siSIRPα LNP treatment trended toward reversed carboplatin resistance in OvCa/macrophage heterospheroids. Where control heterospheroids maintained 53.16 ± 1.21% viability in the presence of 250 μM carboplatin, LNP treatment decreased that viability to 46.37 ± 0.85% (****p < 0.0001, two-way ANOVA, Figure 5B). After 500 μM chemotherapy, siSIRPα LNP-treated heterospheroids were still 3.64% less viable (*p < 0.05, two-way ANOVA, Figure 5B), resulting in an improved IC_50_ of 320.7 ± 54.57 μM compared to 368.2 ± 37.70 μM with no siSIRPα treatment (ns, one-way ANOVA, Figure 5B). Importantly, siSIRPα LNP treatment did not impact OvCa monospheroid viability or chemosensitivity to carboplatin (Figure S2A,B). Heterospheroids treated with negative control (scramble) siRNA NP also maintained stable viability and IC_50_, indicating there are no negative effects of the vehicle itself (Figure S2C,D). Taken together, these data suggest the targeted effect of siSIRPα LNP on macrophages within OvCa/macrophage heterospheroids, impacting platinum chemotherapy sensitivity.

OvCa cells were separated from macrophages following co-culture in heterospheroids by GFP-based fluorescence activated cell sorting. The presence of macrophages in OvCa spheroids increased *MMP-9* signaling indicative of OvCa metastatic potential. This macrophage effect was reduced by blocking CD47-SIRPα signaling with siSIRPα LNP treatment (***p < 0.001, two-way ANOVA, Figure 5D). The *SNAI1* gene was also slightly reduced following siSIRPα LNP treatment (ns, two-way ANOVA, Figure 5D).

Invasive potential of monospheroids and heterospheroids was assessed by transferring spheroids treated with siSIRPα LNPs or maintained as controls into traditional well plates. Spheroids were allowed to attach to the plates, and the area occupied by cells as they migrated away from the spheroid was measured over 5 days (Figure 5E). Macrophages increased OvCa migration by more than 1.5 times (compare 39.34 ± 3.23-fold increase in monospheroid area to 61.17 ± 7.73-fold in heterospheroids, ****p < 0.0001, two-way ANOVA, Figure 5E). Macrophage-driven migration was partially reversed when siSIRPα LNPs were used to inhibit CD47-SIRPα signaling in the spheroids prior to evaluation of migration (ns, two-way ANOVA, Figure 5E).

## 4 Discussion

More than 80% of OvCa is already metastatic at the time of diagnosis.^47^ Metastatic OvCa is more invasive and resistant to chemotherapy than localized disease giving rise to survival rates of grave concern.^48^ Once the OvCa cells exfoliate from the primary tumor, they aggregate within the malignant ascites, where they interact with many other stromal cells, including macrophages. Our previous work and well-established literature have demonstrated that OvCa/macrophage interactions drive carboplatin chemoresistance and invasiveness, contributing to malignant progression. Therefore, in our current studies, we chose to focus on these functional aspects of OvCa malignant progression while evaluating a new therapeutic paradigm.

In the context of OvCa/macrophage interactions, the macrophage checkpoint CD47-SIRPα has emerged as an attractive therapeutic paradigm.^27,30^ Overexpression of OvCa CD47 has been linked to metastasis and devastating prognosis.^27,28,49,50^ Not only do patient samples of advanced OvCa demonstrate positivity for CD47-SIRPα staining (Figure 1B), but heterospheroids bioengineered from the cell line OVCAR3 combined with THP-1 macrophages also robustly express CD47-SIRPα (Figure 1D).

In the CD47/SIRPα-expressing heterospheroid model, reciprocal interactions between the OvCa cells and macrophages drive carboplatin chemoresistance (Figure 2). Heterospheroids generated by coculturing OvCa cells and macrophages were 2.5 times less proliferative than OvCa cells alone (Figure 2B). Macrophages, being the terminal result of monocytic differentiation, lose their ability to proliferate which would account for this reduction.^20,51^ Our work demonstrated that the presence of macrophages decreased OvCa sensitivity to platinum chemotherapy by more than 1.5 times, indicating their contribution to a chemoresistant cancer phenotype (Figure 2C). This finding agrees with the body of research connecting macrophages, via a variety of signaling pathways, to OvCa chemoresistance and ultimately metastasis.^17,19−24,52−55^

CD47 binding to macrophage SIRPα contributes to OvCa progression, making it an attractive therapeutic axis to target, specifically with anti-CD47 antibodies which have historically been studied for disrupting CD47-SIRPα signaling.^29,30,56^ In fact, the first in-human, first-in-class CD47 antibody had positive partial results in prolonging survival of patients with advanced OvCa.^57^ However, CD47 is highly expressed on other cell types, making this class of therapeutics particularly difficult to control in terms of treatment specificity and the resulting adverse effects.^32,36^ CD47 blockade in erythrocytes, for example, causes severe anemias and other drastic blood cell decreases in a vast majority of patients receiving these antibodies, exposing a need for a shift in therapeutic focus.^36^

An alternate way to target the CD47-SIRPα axis is to knock down the expression of macrophage SIRPα using short interfering RNA (siRNA). Liposomes and other lipid nanoparticles (LNPs) have been used clinically for the therapy of cancer and infectious diseases for more than 25 years.^58,59^ In fact, they are the most frequently used nonviral vector for RNA delivery^60^ due to efficient RNA binding and condensing, protection from enzymatic and chemical degradation in the extracellular milieu, and efficient intracellular delivery. Thus, clinically used siRNA therapeutics are based on LNPs.^61^ LNPs are formed by the self-assembly of RNA, phospholipids, and cholesterol, with ease of manufacture and tight quality control (Figure 3). An additional benefit of nanotherapy for this precise application leverages the intrinsic phagocytic property of macrophages. Nanoparticles, as circulating solid particles foreign to the body, have a high efficiency for specifically localizing into macrophages due to the cells’ key role in uptake, processing, and clearing of such substances.^41,62^

Therefore, we chose to take advantage of the natural affinity of LNPs for macrophages to direct an siRNA-based knockdown of macrophage SIRPα. SIRPα siRNA (siSIRPα) LNPs had a diameter around 60 nm with zeta potential measuring approximately −4 mV (Figure 3A,B). These properties are considered desirable based on a variety of studies indicating maximum cellular uptake of particles 40–60 nm in diameter.^63−65^ Additionally, a neutral zeta potential (range −10 to 10 mV) can lead to better stability and increased half-life of particles in circulation, creating a higher chance of intact delivery to the target tissue.^63,66^ The particles efficiently and consistently encapsulated siSIRPα while maintaining uniform particle characteristics over the experimental timelines (Figure 3).

The uptake of the fluorescently labeled siSIRPα LNPs was evident (Figure 4A) in naïve M0 macrophages. Alternatively activated macrophages more typical to the OvCa tumor microenvironment^17,19,20^ were additionally evaluated for siSIRPα LNP uptake. In fact, these alternatively activated macrophages also took up LNPs and did so at rates similar to those of inflammatory M1-like macrophages (Figure S1A,B). Subsequently, uptake of siSIRPα LNPs led to significant changes in macrophage reprogramming of alternatively activated macrophages, evident by increases in gene expression of inflammatory *NOS2*, *IL-1β*, and *IL-12* with a concomitant decrease in gene expression of *IL-10* and *CD206* (Figure S1C). Such macrophage reprogramming has been reported previously with the disruption of the CD47-SIRPα macrophage immune checkpoint axis.^33,67^

In order to evaluate our siSIRPα LNP therapeutic strategy, we utilized the *in vitro* 3D bioengineered model of OvCa/macrophage heterospheroids, focusing on malignant progression readouts like carboplatin chemoresistance and invasiveness. siSIRPα LNP treatment reduced the expression of SIRPα in OvCa/macrophage heterospheroids by ∼50% (Figure 5A). As expected, siSIRPα LNP treatment led to increased sensitivity (lowered chemoresistance) to carboplatin treatment (Figure 5B) likely due to macrophage reprogramming. Disrupting CD47-SIRPα signaling further influenced the OvCa metastatic phenotype by reducing metastasis-indicating *MMP-9* gene expression and cellular invasive potential in our system (Figure 5D,E). These data indicate the relevance of specifically manipulating this macrophage-induced OvCa metastasis.

OvCa CD47 signaling to macrophage SIRPα directly inhibits the anticancer response of macrophages. By definition, this should permit OvCa survival in the presence of phagocytic macrophages. However, our results indicate not only survival but increased OvCa metastatic signatures as a result of OvCa/macrophage interactions, in line with reports in the literature demonstrating anticancer activity upon targeting the CD47-SIRPα axis. Our data strongly indicate the possibility of a new way to disrupt this trophic axis to improve patient outcomes in advanced metastatic ovarian cancer.

## 5 Conclusions

Many prior discoveries have indicated the role that macrophages and the CD47-SIRPα signaling pathway play in OvCa progression. Our hanging drop co-culture spheroids uniquely model the ascites transport of metastatic tumor clusters and situate us to isolate and study the relevant cellular interactions. We leveraged this platform to develop a novel therapeutic strategy localizing to macrophages with lipid nanoparticles specifically designed to safely deliver siRNA to phagocytic macrophages in the tumor microenvironment and reduce SIRPα expression. These findings have the potential to influence advanced therapeutics targeting metastatic OvCa in the clinic to improve therapy response and ultimately patient survival.

## Supporting Information Available

Polarization of M2 macrophages by siSIRPα LNPs, additional controls for LNP-dependent spheroid proliferation, and chemotherapy response (PDF)
