# Supplementary material for: Macrophage Checkpoint Nanoimmunotherapy Has the Potential to Reduce Malignant Progression in Bioengineered In Vitro Models of Ovarian Cancer
Source: ACS Appl Bio Mater. 2024 Apr 1;7(12):7871–82. doi: 10.1021/acsabm.4c00076 (PMC11653402; doi:10.1021/acsabm.4c00076)
Supplement: Supplementary file 1 — mt4c00076_si_001.pdf [file mt4c00076_si_001.pdf]

Supporting Information

Manuscript: Macrophage Checkpoint Nanoimmunotherapy Has the Potential to Reduce Malignant Progression in Bioengineered *In Vitro* Models of Ovarian Cancer

Sabrina N. VandenHeuvel[1#], Eric Chau[2#], Arpita Mohapatra[1], Sameera Dabbiru[1], Sanjana Roy[1], Cailin O'Connell[2,3], Aparna Kamat[4,5,6], Biana Godin[1,2,5,6#], Shreya A. Raghavan[1#]*

[1]Department of Biomedical Engineering, Texas A&M University, 3120 TAMU, College Station, Texas 77843, United States
[2]Department of Nanomedicine, Houston Methodist Research Institute, 6670 Bertner Avenue, Houston, Texas 77030, United States
[3]School of Engineering Medicine, Texas A&M University, 1020 Holcombe Boulevard, Houston, Texas 77030, United States
[4]Division of Gynecologic Oncology, Houston Methodist Hospital, 6550 Fannin Street, Houston, Texas 77030, United States
[5]Department of Obstetrics and Gynecology, Houston Methodist Hospital, 6550 Fannin Street, Houston, Texas 77030, United States
[6]Houston Methodist Neal Cancer Center, 6445 Main Street, Houston, Texas 77030, United States

[#]SNV and EC contributed equally to the manuscript, BG and SAR are shared senior authors

*Address Correspondence to:
Shreya A. Raghavan, PhD (she/her)
5016 Emerging Technologies Building
3120 TAMU
College Station, Texas 77843
Email: sraghavan@tamu.edu
Phone: 979-458-3126

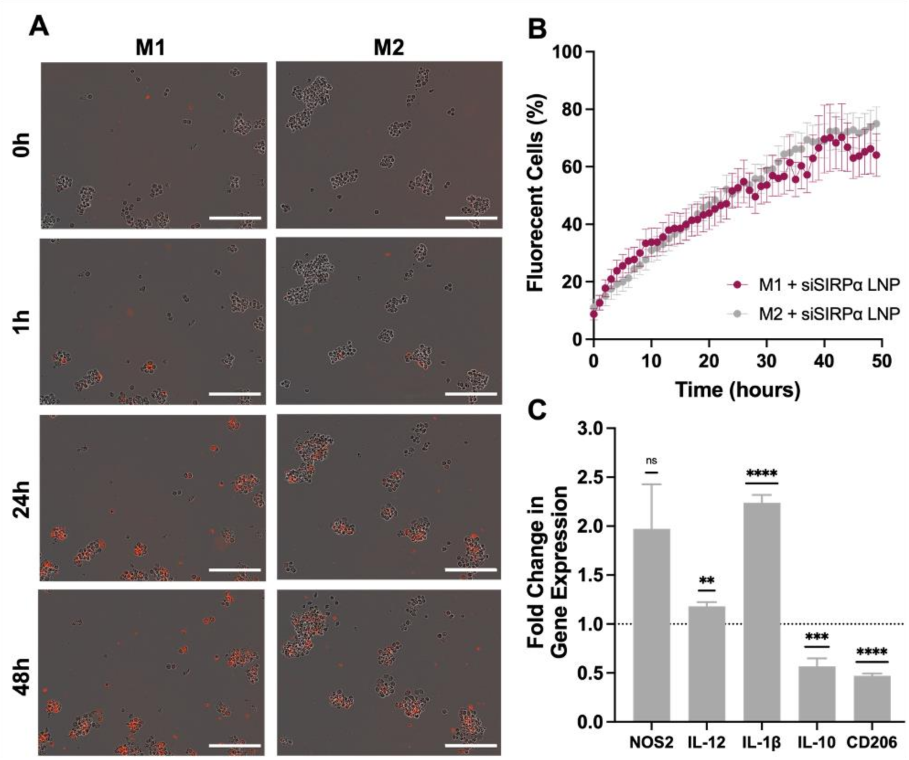

Figure S1. siSIRPα LNP uptake by M1 and M2 macrophages. The immunosuppressive nature of the OvCa tumor microenvironment can drive macrophage polarization toward alternative activation. As such, we tested the ability of such a macrophage population to uptake siSIRPα LNP and the subsequent effect of the LNP on macrophage phenotype compared to an anti-tumoral (M1) phenotype. (A) THP-1-derived macrophages were polarized to M1 or M2-like phenotypes with IFNγ (50 ng/mL)/LPS (25 ng/mL) or M-CSF (50 ng/mL)/IL-4 (50 ng/mL), respectively. Both M1 and M2 macrophages efficiently took up fluorescently labeled siSIRPα LNP as indicated by red signal in live-cell images taken over 48 h after LNP administration. Uptake continued over 48 h with increased red fluorescence visible with time. Scale bars = 200 µm. (B) Image quantification confirmed this uptake efficiency showing that >50% of cells exhibited fluorescence by 30 h. This data indicated not only that macrophages which have likely been pushed to an M2 phenotype in the OvCa tumor microenvironment successfully uptake the nanoparticles, but also that, upon CD47-SIRPα blockage, the phagocytic ability of M1 and M2 macrophages present similarly. This could indicate a shift in phenotype due to LNP treatment. (C) To evaluate whether this phagocytic activity was due to a shift in polarization, gene expression analysis was performed on M2 macrophages treated with siSIRPα LNP. LNP did induce a more M1-like gene signature in the M2 macrophages with significant increases in *IL-12* and *IL-1β* (**p=0.001, ****p<0.0001, respectively, one sample t test) paired with corresponding reduction in *IL-10* and *CD206* M2 expression (***p<0.001, ****p<0.0001, respectively, one sample t test).

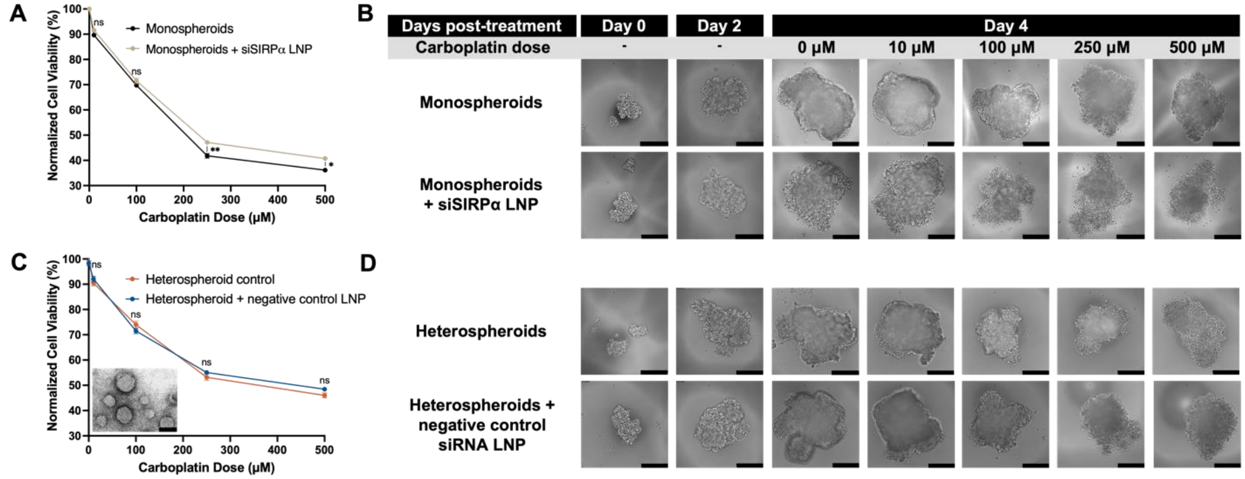

Figure S2. Carboplatin sensitivity in control spheroids. siSIRPα LNP treatment was shown to slow growth and decrease viability in chemotherapy-treated heterospheroids. To confirm whether this was a toxic effect of the LNP or rather OvCa growth reduction resulting from CD47-SIRPα inhibition, we tested additional controls in the presence of LNP. (A) OvCa monospheroids were similarly responsive to carboplatin with and without siSIRPα LNP treatment (compare IC_{50} value of 218.1 μM in control spheroids to 272.1 μM after siSIRPα LNP). As hypothesized, LNP did not appear to have a toxic effect on the OvCa cells as evidenced by this maintenance and even slight increase of cell viability (5.34% higher at 250 μM, **$p<0.002$; 4.57% higher at 500 μM, *$p<0.02$, two-way ANOVA). (B) Further, phase contrast micrographs show comparable spheroid size and morphology after 2 days of LNP treatment and after another 2 days with 0-500 μM chemotherapy confirming the LNP are not acting on OvCa cells to reduce viability. Scale bars = 200 μm. (C) LNP containing negative control (scramble) siRNA were administered to OvCa/macrophage heterospheroids to evaluate the potential adverse effects of the LNP vehicle on spheroid viability. A transmission electron micrograph (50,000X; scale bar = 50 nm) illustrates the uniform size distribution of negative control LNP. Heterospheroids did not exhibit any signs of toxicity in response to the negative control LNP as evidenced by similar IC_{50} values (368.2 μM with no treatment and 405.3 μM with scramble LNP, ns, one-way ANOVA), no changes in cell viability at any one dose (ns, two-way ANOVA), and (D) comparable phase contrast micrographs (scale bars = 200 μm).
